# Social entrepreneurship in obesity prevention: A scoping review

**DOI:** 10.1111/obr.13378

**Published:** 2021-11-28

**Authors:** Audrey Chia, Junyu Ong, Anjali Bundele, Yee Wei Lim

**Affiliations:** ^1^ NUS Business School National University of Singapore Singapore Singapore; ^2^ Yong Loo Lin School of Medicine National University of Singapore Singapore Singapore; ^3^ Duke‐NUS Medical School National University of Singapore Singapore Singapore; ^4^ Alexandra Hospital Singapore Singapore

**Keywords:** obesity, obesity prevention, public health, social entrepreneurship

## Abstract

We conducted a scoping review of social ventures in obesity and developed a taxonomy of their interventions and business models. Sources included PubMed, Business Source Premier, ABI Inform, Factiva, Google, Facebook, Twitter, social entrepreneurship networks (Ashoka, Skoll, and Schwab), and social entrepreneurship competitions. Our review identified 512 social ventures in 32 countries; 93% originated from developed countries. Their areas of intervention included diet and nutrition, urban farming, physical activity, access to healthy food, and health literacy. They addressed factors beyond health such as education, affordability, employment, and the built and natural environments. To support their programs of work, social ventures developed various business models with multiple revenue or resource streams. Social ventures designed double‐duty interventions that were aligned with additional meaningful social or environmental objectives. This “bundling” of objectives allowed social ventures to appeal to a wider target audience. Most of the social ventures were initiated, supported, or sustained by local communities. Social ventures offer financially self‐sufficient approaches to obesity reduction and could potentially relieve the burden on healthcare systems. Policymakers should consider social entrepreneurs as partners in obesity prevention.

**Abbreviations**
US: United States of America
WHO: World Health Organization

## 1 INTRODUCTION

Despite the rising prevalence of obesity and its associated socioeconomic costs, effective strategies against it remain elusive. A Cochrane review update (2019) on obesity interventions reported that diet and physical activity interventions produced only modest weight reduction in children aged 12 and below.^1^ Among adolescents aged 13 to 18, there was no strong evidence that these obesity interventions were effective for weight reduction.^1^ Among adults, systematic reviews in 2018 and 2019 on nonsurgical obesity interventions reported limited effectiveness in weight loss maintenance.^2^, ^3^, ^4^ The US National Weight Control Registry data indicated that 80% of overweight participants regained their weight within a year.^5^

In the last 30 years, no country has managed to reverse the rise in obesity; all 200 World Health Organization (WHO) member countries are unlikely to meet targets to halt the rise in obesity by the year 2025.^6^, ^7^ Among 140 lower and middle‐income WHO member countries, only 54 (38%) had national policies to reduce obesity; these focused more on individual consumers and government agencies and less on businesses and civil society.^8^ Obesity has multiple causes: genetics, individual behavior, and physical and social environments. It calls for multipronged and novel approaches that include a range of stakeholders.^9^ Among such stakeholders are social entrepreneurs.

In recent decades, social entrepreneurs have emerged where governments and markets have failed to meet basic needs for water, sanitation, primary healthcare, or specialist care.^10^ In our earlier research, we noted that social entrepreneurs share a common set of practices, applying business and entrepreneurial approaches to solve social problems of disadvantaged populations.^11^ Social entrepreneurs are not a highly codified profession, but there is a set of practices that typify social entrepreneurship, as elaborated below.^12^, ^13^

Unlike charities, social entrepreneurs do not typically rely on donations or aid as their main source of funding. Social ventures aim to become self‐sufficient by developing viable business models and multiple streams of financial and nonfinancial support. Social entrepreneurs often engage with the communities that they aim to serve, involving them as partners or codesigners of solutions. Because they interact with different groups of stakeholders, social entrepreneurs are likely to view problems in context and acknowledge the interrelations between health and nonhealth factors, for example, between lack of education and health‐seeking behavior, or poverty and ill‐health. Rather than providing products and services for free, social entrepreneurs develop frugal solutions to deliver healthcare and social services. The dual focus on affordability while empowering or educating communities allows social entrepreneurs to sustain their programs of work.

The organizations founded by social entrepreneurs are called social ventures, in recognition of their risk‐taking and entrepreneurial spirit. As with other business ventures, social ventures may fail. The failure rate may be even higher among social ventures than in mainstream business ventures, because they often take on challenges where there have been government or market failure. In most countries, social ventures do not have a distinct business registration category. They can be registered as for‐profit or nonprofit entities, or hybrid entities with separate for‐profit and nonprofit structures. There are striking examples of social ventures that have successfully innovated to meet diverse health and medical needs: rural health (Population and Community Development Association Thailand), water and sanitation (World Toilet Organization), ophthalmology (Aravind Eye System), and cardiovascular surgery (Narayana Health). We chose to focus on social ventures because strategies against obesity require community involvement or a “whole of society” approach.^14^, ^15^

Our primary objective was to conduct a scoping review of social ventures in obesity. We sought to understand the work of these social ventures by developing a taxonomy of:

(a) What they did: their interventions,
(b) Where they worked: their target populations and location of interventions; and
(c) How they supported their work: their business models.

## 2 METHOD

Our global scoping review of social ventures in obesity covered academic and nonacademic databases, news databases, and web‐based searches of Google and social media. Our goal was not to find all obesity‐related social ventures but to identify relevant and representative examples, and then develop a taxonomy of their obesity interventions, contexts, and business models.

### 2.1 Information sources

Our sources included academic and news databases (PubMed, Business Source Premier, ABI Inform and Factiva), social entrepreneurship networks (Ashoka, Skoll Foundation, and Schwab Foundation), social media (Facebook and Twitter), and social entrepreneurship competitions across the United States, Europe, and Asia. We also performed Google searches. From academic databases, we identified articles, dissertations, and working papers. From Factiva, we accessed 32,000 news sources, including newspapers, magazines, television, and radio transcripts. The inclusion of unconventional data sources allowed us to search more widely for social ventures in obesity.

### 2.2 Database search strategy

The objective of the search was to identify social ventures working in obesity. We searched for social ventures that addressed obesity or determinants of obesity such as diet, physical activity, and healthy food sources.

The search terms included social entrepreneurship and its related concepts (social innovation, social enterprise, and social venture). For “obesity,” we used terms related to both prevention and treatment (physical activity, exercise, diet, nutrition, healthy eating, workplace health, weight loss, weight gain, ideal weight, urban farming, and indoor farming). Our search terms acknowledged obesity‐relevant contexts. “Workplace health” was included because people spend a significant proportion of their waking hours at work. “Urban farming” and “indoor farming” were included because such farms are part of the food ecosystem. Urban farms address multiple determinants of obesity by diversifying diets, improving access to fresh and healthy food and encouraging physical activity.

Combining search terms related to both obesity and social entrepreneurship related yielded 28 unique search strings that were modified to match each database's search syntax. The search included truncation or asterisk wildcards for each phrase, where necessary. For example, in academic and industry databases, we used “social entrepreneur*” AND “obesity”; for Google searches, we combined “social entrepreneur” AND “obesity.” For social media searches, in Facebook, we used (social entrepreneur) AND (obesity); and in Twitter, #socent and #obesity (see the supporting information for more details).

Articles dating from January 1980 to March 2020 were gathered from PubMed, Business Source Premier, ABI/Inform, and Factiva. Google searches were performed between August to September 2019 and in March 2020. We reviewed the first 10 pages of each Google search; beyond this, hits became redundant. In Facebook, we used Advanced Search, and filtered only for pages of organizations. On Twitter, we used Advanced Search of keywords with the option “All these words.” Additional searches were done for obesity‐related terms and the hashtag “#socent.” From social entrepreneurship competitions in the United States, Europe, and Asia from 2015 to 2020, we reviewed finalists working on obesity. We searched the directories of Ashoka,^16^ Schwab Foundation for Social Entrepreneurship, and Skoll Foundation, three organizations that promote, recognize, and support social entrepreneurs globally. Paid web links were excluded. Each social venture was checked for existence and legitimacy using its official website and other Google searches.

### 2.3 Criteria for inclusion

To be considered for inclusion, a social venture had to have at least one revenue or resource stream that partly or entirely sustained its work, and not solely or mostly provide free products and services. These two criteria were based on key characteristics of social entrepreneurship and helped distinguish social ventures from charities and welfare organizations that relied mostly on donations. We included social ventures with activities that were commonly associated with obesity prevention. Social ventures that engaged in any of these activities individually or in combination were included. Among our social ventures were those that advocated or promoted physical activity by delivering products or services (e.g., specialized exercise equipment or fitness coaching) or by providing venues or infrastructure for physical activity. Other ventures encouraged the consumption of healthy food; they ran restaurants, kitchens, and other initiatives that provided healthy meals or instruction in cooking or nutrition. Yet other ventures increased access to healthy food (e.g., mobile food markets) or improved affordability, or boosted its supply (e.g., greenhouses).

We excluded entities without an explicit social purpose, because social ventures are defined by having a social purpose as their main objective. Hence, entities that did not explicitly mention in their websites, social media, or annual reports that they had a social purpose were excluded.

We also excluded ventures for which there were no data or record of performance, for example, early stage ventures in incubation that had no business activity, ventures that had ceased operations, and ventures with insufficient information (no website, website not in English, or incomplete information). As our search was wide‐ranging, our social media search results sometimes listed organizations that worked in obesity but were not social ventures. We excluded these from the review: government entities, research trials/interventions, educational institutions (except as partners), foundations that funded social ventures but were not otherwise engaged in any social entrepreneurial activities, and healthcare institutions (unless the explicit aim was to address obesity).

### 2.4 Data items and synthesis of results

We extracted the following information on each social venture: country of origin, business model, years in operation, category of obesity intervention (such as diet and nutrition, promoting physical activity), setting of obesity intervention, target beneficiaries, and impact reporting. A complete list of data items is shown in the supporting information.

We reviewed the official websites of all identified ventures. Additional information was obtained from each venture's social media sites, press releases, and financial reports, if available. All ventures identified were independently reviewed by JY and AMB using the inclusion and exclusion criteria. Entities that met the criteria were collated into a master database of social ventures. LYW and AC adjudicated any disagreements between JY and AMB. All differences were resolved by consensus.

**FIGURE 1:**
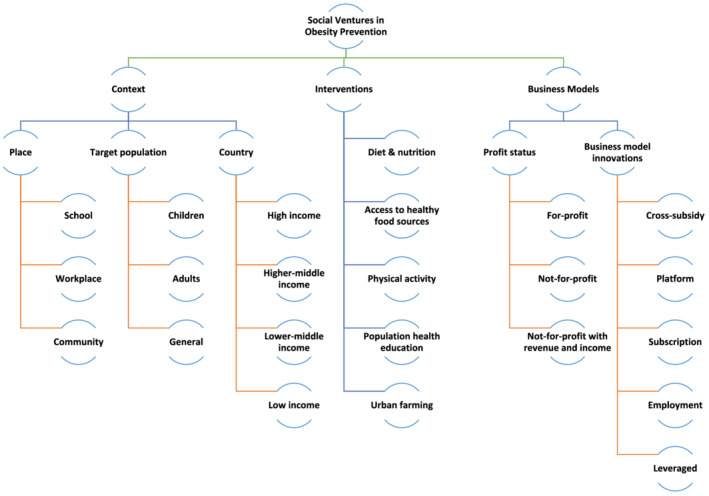
Classification of social ventures in obesity

We developed a taxonomy of the social ventures in obesity using an iterative approach (see Figure 1). As mentioned in our criteria for inclusion, only social ventures—those with an explicitly stated social purpose—were included. Second, social ventures that worked primarily in health were distinguished from those that did not. Among health ventures, we identified those working on obesity. Obesity‐related ventures were then classified according to their interventions, target population, and business models. While business models and target populations had clear, preexisting classifications, the classification of obesity interventions was developed using an iterative approach based on the information about each venture.

We examined the relationship between types of interventions (i.e., diet and nutrition, urban farming, physical activity, access to healthy food sources, and population health education) and settings of intervention (community, school, and workplace). This probed whether certain types of intervention were more commonly found in some settings or target populations. We also examined whether impact reporting was more frequently undertaken by for‐profit or nonprofit ventures. For this analysis, group differences were evaluated with a chi‐square test. Statistical significance was set at *p* < 0.05.

## 3 RESULTS

### 3.1 Selection of social ventures

**FIGURE 2:**
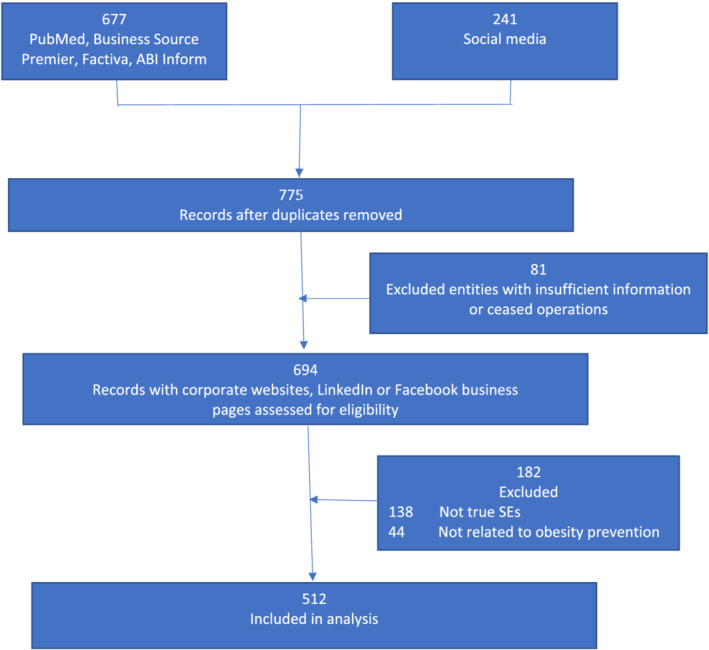
Flowchart for inclusion of records

Our search identified 918 social ventures for possible inclusion. Excluding duplicates, 775 ventures remained. Another 81 were removed due to lack of information or cessation of operations. A further 182 were removed because they lacked social entrepreneurial characteristics or were unrelated to obesity. The remaining 512 social ventures met all criteria for inclusion (Figure 2).

### 3.2 Context of operation

Of the 512 social ventures, 93% were founded in high‐income countries and 7% in upper‐middle and lower‐middle‐income countries (World Bank Income Classification 2020). The top five countries of origin were the United States (56%), United Kingdom (19%), Canada (6%), Australia (5%), and India (2%). Almost all (97%) the social ventures operated only in their country of origin. The average number of years in operation was 16.4 years, with a median of 12 years.

In addition to obesity, almost half (45%) the social ventures in our review had social and nonhealth objectives unrelated to obesity. These diverse objectives included livelihoods for refugees, promotion of environmental business practices, and advocacy for women's rights. Four‐fifths (81%) of social ventures worked to strengthen their local communities by capacity building, education, and other means.

### 3.3 Interventions in obesity

#### 3.3.1 Categories of intervention

We identified five categories of intervention: diet and nutrition (59%), urban farming (43%), physical activity (31%), access to healthy food sources (22%), and population health education (16%) (see supporting information for details). In each category, there were diverse and innovative approaches to preventing obesity (see Table 1 for examples).

**TABLE 1:** Categories of obesity interventions by social entrepreneurs, with examples

Category of Intervention	Social Venture	Programmes and Activities
**Diet & Nutrition**	**Locol (USA)**	Served healthy fast food at affordable prices Reduced use of processed foods and introduced grains and tofu to burgers; no soft drinks served
	**Xilinat (Mexico)**	Collected agricultural waste, which would otherwise be incinerated, from farmers Invented patented fermentation process to transform agricultural waste into xylitol, a low‐calorie sugar substitute
	**Bollywood Veggies (Singapore)**	Taught children culinary skills Served local cuisine prepared from ingredients sourced directly from the farm
**Physical Activity**	**The Oopoeh Foundation (Netherlands)**	Connected busy or travelling dog owners to senior citizen volunteers for pet sitting services. Increased physical activity and reduced social isolation among senior citizens.
	**Siel Bleu (France)**	Provided specialised physical training in healthcare institutions for the elderly, people with disabilities, and patients with chronic medical conditions
	**KaBOOM! (USA)**	Built and refurbished play spaces for children and youth, especially in low‐income neighbourhoods
**Population Health Education**	**Food Heroes(China & USA)**	Provided nutritional education training for teachers Provided lesson plans which can be integrated into existing school curriculum for children aged 5 to 9.
	**Healthy School Food Collaborative (USA)**	Provided consulting, vendor and procurement services to schools with child nutrition programs Trained school staff and monitor implementation of school food programs
	**ProAge (Singapore)**	Provided consulting on workplace wellness Worked with corporate clients to conduct workplace fitness training, health and safety screening
**Urban Farming**	**Rooftop Republic (Hong Kong)**	Partnered architects and property developers to build urban rooftop farms Leveraged on unutilised urban space in land‐scare Hong Kong to grow fresh produce sold to local restaurants
	**Fresh Roots (Canada)**	Partnered the Vancouver School Board to run educational farms in various schools Food grown was sold at school cafeterias and to local residents Offered internships to students to grow, harvest and prepare meals for the community
	**Fresh Future Farm (USA)**	Operated an urban farm and grocery store in a minority neighbourhood with poor access to fresh produce Provided employment and/or training for new farmers
**Healthy Food Sources**	**Oddbox (UK)**	Purchased fresh produce deemed not saleable from local farmers and supermarkets Resold fresh produce in sustainable packaging for home and office deliveries Surplus produce donated to charities
	**Community Foods Market (USA)**	Operated a supermarket in a low‐income neighbourhood with little access to fresh produce Enabled residents to pay with food stamps and offer discounts to senior citizens Ran nutritional classes and blood pressure clinics for the public
	**Fresh International Gardens (USA)**	Hired and trained refugees to farm in Alaskan climate Integrated refugees into local community through language training, and holding farmers’ markets for them to sell produce directly to local residents Reduced local dependence on imported fresh produce

Social ventures typically developed interventions in more than one category. Bollywood Veggies, a Singapore‐based venture, organized physical activities such as rice planting for adults and conducted healthy cooking classes for children.^17^ Community Foods Market, a US social venture, started as a mobile fresh food stand in a food desert neighborhood.^18^ It grew into a supermarket that partnered nongovernmental organizations to run blood pressure clinics and nutrition classes at its premises (see Table 1).

We found social ventures that adopted interventions integrated across the value chain. They typically engaged in stages from farm to table, including agriculture, distribution, and food preparation and retail. Fresh Roots (see Table 1) partnered schools in Vancouver to grow food on school grounds, sell produce to local residents and prepare meals for school cafeterias. Fresh Roots is an example of a venture that created interventions spanning four of our five categories—diet and nutrition, urban farming, physical activity, and access to healthy food sources.

Whereas 68% of ventures did not target a specific age group, 30% targeted children; and 2% targeted older adults. Social ventures operated in local communities (93%), schools (39%), or workplaces (9%). The percentages indicated that some ventures worked in more than one setting. We examined the intersection between categories and settings of intervention. The top three interventions in local communities were diet and nutrition (58%), urban farming (44%), and physical activity (31%). The same top three interventions were found in schools: diet and nutrition (75%), urban farming (48%), and physical activity (33%).

#### 3.3.2 Business models

Two‐fifths (39%) of the ventures were for‐profit, and the rest nonprofit. Almost half (49%) of the social ventures aimed at financial self‐sufficiency, that is, zero‐reliance on donations; 43% of social ventures were already self‐funding. There was a range of business models including cross‐subsidy, subscription, employment, leveraged, and platform models (see Table 2). Good Bowls from North Carolina, USA, used a cross‐subsidy model. They sold the same healthy frozen meal for a lower price in convenience stores in low‐income neighborhoods, and at a higher price in premium food stores. Customers at premium stores could choose to pay US$2 more to subsidize meals for low‐income customers.^19^ Other social ventures used a subscription model to attain a steady stream of income while instilling health dietary habits in customers. For example, Eat My Lunch (New Zealand) offered subscription plans for regular delivery of healthy weekday meals. A platform model such as that of Box Divvy (Australia) allowed customers to buy fresh produce directly from local growers while saving costs, supporting local farmers, and reducing the environmental footprint of food.

**TABLE 2:** Examples of innovative interventions developed by social entrepreneurs

Area of Innovation	Social Ventures	Description
**Urban Renewal**	**Sole Food Street Farm (Vancouver, Canada)**	Reclaimed vacant, contaminated land in city slums into urban farms; produce was sold locally Trained people who recovered from substance abuse and patients with mental health conditions as farmers
**Food Wastage**	**Hungry Harvest (USA)**	Rescued odd‐sized or surplus produce from farmers, packing houses and wholesalers Produce was repackaged and sold to multiple cities under a weekly subscription model
**Combating Obesity and Malnutrition**	**Table for Two (Japan)**	Worked with corporate and school cafeterias in Japan to serve healthy food Directed part of proceeds from each healthy meal purchased towards funding meals for children in developing countries
**Technology**	**PlayCity (Canada)**	Partnered sports centres and recreational facilities to advertise activities on the free‐to‐use PlayCity app Connected users looking for players and venues for physical activities (such as basketball, hockey, bowling, and trampoline)
**Business Model: Subscription**	**Eat My Lunch (New Zealand)**	Offered a subscription‐based, healthy meal service that delivered directly to offices and schools every weekday Customers could also purchase meals for school children from low‐income families
**Business Model: Cross Subsidy**	**Good Bowls (USA)**	Sold healthy frozen meals at different price points; meals cost US$2.99 in convenience stores at low‐income neighbourhoods and US$4.99 at premium food stores Customers at premium food stores had the option to pay an extra US$2 (or US$6.99) to further subsidise meals sold to low‐income consumers
**Business Model: Platform**	**Box Divvy (Australia)**	Connected local farmers to residents in West Sydney via an online platform The online platform model offered farmers better prices compared to negotiating with wholesalers; customers saved on costs by buying directly from farmers
**Business Model: Employment**	**Green Bridge Growers (USA)**	Trained and hired young adults with autism in aquaponic farming
**Business Model: Leveraged Non‐profit**	**Green Urban Lunch Box (USA)**	Trained volunteers in organic farming and connected these volunteers to elderly residents with unused backyards Provided materials for volunteers to build and maintain organic gardens in the elderly residents’ backyards at no cost to them. The produce was shared among volunteers, elderly residents and the social venture.

One third (34%) of social ventures addressed obesity‐related health or social challenges of their target populations. These ventures recognized that obesity could be associated with socioeconomic conditions such as low incomes or other physical conditions such as disability. They served low‐income families (20%); people with medical conditions including diabetes, mental illnesses, and physical disabilities (5%); and people living in food deserts—residential areas with poor access to affordable, nutritious food (4%). Fresh Future Farm managed an urban farm and a grocery store in a minority neighborhood with limited access to fresh fruits and vegetables, in South Carolina, USA (see Table 1).^20^ Parakids from Bulgaria provided specialized sporting equipment and activities to children with disabilities and postural problems.^21^

### 3.4 Impact reporting

Of 512 social ventures, 25% reported the impact of their work. Only 16% reported their impact in the last 2 years. Three‐quarters of the 16% were nonprofit ventures. Only 3% of ventures reported third‐party evaluations.^21^ Most social ventures reported on activities and number served, rather than results, for example, efficacy of services/products in promoting weight loss. Not‐for‐profit ventures were three times more likely than for‐profit social ventures to report their impact in annual reports and websites (*X* ^2^ = 42.05, *p* < 0.001).

## 4 DISCUSSION

This is the first comprehensive scoping review of social ventures in obesity. The social ventures created diverse solutions such as reinventing fast food, developing play spaces for children, and reclaiming vacant, disused land for farming. Many ventures operated in a single location, initiated by residents. The benefits of such an approach include sensitivity to the local culture and context, community involvement, and sustainability. This may also explain the difficulty that social ventures faced in scaling up or replication in other countries. Only 3% of the social ventures operated outside their country of origin. Among them was Green Monday, a Hong Kong‐based social venture that raised US$70 million from private equity funds to launch plant‐based food products across 20 markets, including China and Singapore.^22^ Within Hong Kong, Green Monday also partnered with schools, universities, and companies to add vegetarian options in their cafeterias. Green Monday is more of an exception rather than a typical example, but with the rising interest in plant‐based diets as a response to climate change,^23^ we expect more interest in and support for such social ventures. Like any other kind of business venture, social ventures need a supportive ecosystem with elements such as funding, accelerators, access to advice and social networks, links with private sector companies, and even government support.^24^, ^25^

As obesity is a challenge faced by many developed countries, it is unsurprising that the majority of social ventures in obesity (93%) originated and operated in high‐income, developed countries. However, the low representation among developing countries is a cause for concern. The prevalence of overweight and obesity in developing countries is almost 30%. Nearly two‐thirds (62%) of the world's people with obesity or overweight live in developing countries.^26^ As we noted in Section 1, less than two‐fifths of middle‐ to low‐income countries had policies related to obesity reduction; general attention to obesity might have been affected by this lack of national agenda. In addition, as our other research suggests, public health problems such as malnutrition, maternal and infant health, and poor healthcare delivery take higher priority in developing countries.^11^

Obesity has an immediate impact on a child's health, educational attainment, and quality of life.^27^ Children living with obesity are likely to do so as adults and risk developing noncommunicable diseases.^28^ Social ventures in our review seem to have recognized these facts, as more than a third of them focused on obesity among children, and implemented interventions in schools. It might be worth exploring how schools could collaborate with social entrepreneurs in their efforts to prevent obesity.^29^

More than half of the world's population live in cities, and the United Nations has estimated that the proportion will increase from 55% in 2018 to 68% in 2050.^30^ It is appropriate therefore that obesity interventions should also target the problems or opportunities afforded by an urban setting. The second largest proportion of ventures in our review worked on urban farming. Despite entry barriers such as the cost of land and resources, urban farming proved attractive as a holistic strategy. Urban farms improved access to fresh produce, encouraged outdoor time and physical activity, and provided community‐based education about nutrition, ecology, and sustainability.^31^, ^32^, ^33^ We expect urban farming to increase in popularity not only for reasons related to health and climate but also for food security.

Almost half the social ventures took what the Lancet Commission termed “double‐duty or triple‐duty actions”.^6^ They simultaneously addressed obesity and other problems such as employment and poverty. For example, Sole Food Streets Farm transformed vacant, contaminated land in Vancouver slums into urban farms and trained patients with chronic mental health conditions as farmers.^34^ Table for Two—which originated in Japan and has since expanded to Korea, the United States, and Germany—sold balanced meals and donated the cost of calories saved to meals for undernourished students in Africa and the Philippines. Both examples illustrate multi‐duty interventions that addressed obesity along with other positive social goals.

Double‐ or triple‐duty interventions that blended obesity with other social objectives increased the appeal of social ventures' products and services. Besides marketing their fresh vegetables as contributors to healthy diets and lifestyles, Sole Food Street Farm and Hungry Harvest emphasized that they reduced food waste and supported local employment (see Table 2). Similarly, Table for Two explained how each healthy meal subsidized a meal for a malnourished child. Only 15% of the social ventures explicitly stated obesity prevention as a primary goal. This could be partly due to the stigma attached to obesity and weight. The majority chose positive social messaging. The approach of “bundling” healthy eating with positive social impact enhances the attractiveness of each.^22^ Such “bundling” could also assist policymakers in advocating healthier lifestyles to the public.

More than two‐fifths of the social ventures that we reviewed had become self‐sustaining. These for‐profit or nonprofit ventures sustained their operations by developing multiple streams of financial and nonfinancial support while addressing social goals. Our review provides evidence that whether they are for‐profit or nonprofit, social ventures in obesity can become viable and not have to rely on donations or public funding to sustain their work. This makes social ventures, despite their risk of failure, an attractive complementary mode of addressing obesity.

The low incidence of impact reporting among social ventures in this review can be partly explained by limited time and resources. Social ventures have to choose between spending time and effort to manage and grow their programs of work, or to evaluate and report impact. Another reason is that social ventures' interventions do not exist in a vacuum. It is not a trivial effort to objectively assess the impact on community health while accounting for diverse, confounding variables such as educational attainment, family income, urban environment, and local government policies. Resource‐intensiveness and research challenges may explain the low incidence of evaluation and impact reporting. However, donors and third‐party investors do expect impact reports as part of stakeholder accountability^35^; this may account for the higher frequency of reporting among nonprofit social ventures. Among social ventures that have been recognized or awarded, due diligence has been done by the awarding organizations, such as Ashoka, Skoll Foundation, and Schwab Foundation.^36^ Social venture competitions also investigate their candidates.For example, Clinicas del Azucar, which was in our review, was awarded first prize in the Swiss Re Entrepreneurs for Resilience Award 2021. The Swiss Re Foundation carefully assesses the track record of candidates and performs on‐site checks.^37^ These third‐party checks give us some confidence that although the rate of public impact reporting is low, many of the ventures in our study have undergone and passed scrutiny by external organizations.

### 4.1 Strengths and limitations

To our knowledge, this is the first scoping review of social ventures and their obesity interventions. Our broad search that included unconventional web‐based sources of information and gray literature enabled us to better capture examples beyond the scope of the academic literature. We believe that the social ventures we have identified are representative of the state of social entrepreneurial practice in obesity prevention.

As the search was conducted in English, most of the social ventures that we included had English corporate websites or business profiles. Eight percent of social ventures originated from countries where English was not predominantly used. We acknowledge that we would probably have had wider representation if our search had been conducted in other languages. Our Google searches tended to yield results that were more locally relevant, even after we disabled the location of the computer. This was because the Internet service provider was located in Singapore. It was unfeasible for us to obtain a Virtual Private Network to various countries to run more searches.

## 5 RECOMMENDATIONS

We hope that this review will motivate governments and healthcare systems to support social ventures in obesity prevention and other areas of health. As with other business ventures and innovations, social ventures carry a high risk of failure, but the ones that have survived represent an alternative, community‐based means of addressing obesity. As we noted above, impact reporting by social ventures should be encouraged and supported. Funders or social venture networks could provide training for social ventures in evaluation and reporting.

From a public health research perspective, we hope more evaluation research of social ventures' programs could be conducted to assess their impact and sustainability. Social entrepreneurs and policymakers would benefit from knowing what interventions affect obesity‐related outcomes and which operational models are feasible across communities with different social contexts and levels of resource availability.

Collaborations with established social ventures could complement policymakers' attempts to address obesity and related social and environmental challenges. Finally, policymakers could adopt social entrepreneurial thinking and practices as they seek innovative solutions to the stubborn problem of obesity.

## 6 CONCLUSION

The battle against obesity has been long and difficult, because the problem is complex.

It needs to be fought across settings (work, school, or home), sectors (government, business, and civil society), and levels (individual, societal, and national).^14^

Our review found that social ventures implemented a diverse range of obesity‐related interventions across different contexts. They addressed obesity together with other health and nonhealth objectives, and aligned obesity reduction with appealing causes such as environment and poverty alleviation. Many were owned by local communities, sustained by multiple streams of resources, and developed business models to sustain their work. They indicate that obesity interventions can become financially self‐sufficient and potentially alleviate the burden on healthcare systems and budgets. Our review provides multiple glimpses of what the Lancet Commission calls “sustainable, profitable models that explicitly include benefits to society and the environment.”^6^

## CONFLICT OF INTEREST

No conflict of interest statement.

## FUNDING INFORMATION

This study was funded by the War on Diabetes Seed Fund administered by Saw Swee Hock School of Public Health, National University of Singapore.

## Supporting information


**Data S1.** Supporting information
