# Supplementary material for: Social entrepreneurship in obesity prevention: A scoping review
Source: Obes Rev. 2021 Nov 28;23(3):e13378. doi: 10.1111/obr.13378 (PMC9285950; doi:10.1111/obr.13378)
Supplement: Supplementary file 1 — Data S1. Supporting information [file OBR-23-0-s001.docx]

# Supplement

## Database Search Terms

| Database and Search Terms [Search performed between 1 Aug 2019 and March 2020] |
| --- |
| **ABI INFORM** |
| "Social Enterprise*" AND "farm*" |
| "Social Enterprise*" AND "indoor farm*" |
| "Social Enterprise*" AND "physical activity" |
| "Social Enterprise*" AND "urban farm*" |
| "Social Enterprise*" AND "weight loss" |
| "Social Enterprise*" AND Diet |
| "Social Enterprise*" AND Obesity |
| "Social Entrepreneur*" AND "Indoor Farm*" |
| "Social Entrepreneur*" AND "Physical Activity" |
| "Social Entrepreneur*" AND "Urban Farm*" |
| "Social Entrepreneur*" AND Farm* |
| "Social Entrepreneur*" AND Obesity |
| "Social Entrepreneur*" AND "Weight Loss" |
| "Social Entrepreneur*" AND Diet |
| "Social Innovat*" AND "Farm*" |
| "Social Innovat*" and "Indoor Farm*" |
| "Social Innovat*" AND "Physical Activity" |
| "Social Innovat*" and "Urban Farm*" |
| "social innovat*" AND "weight loss" |
| "social innovat*" AND diet |
| "social innovat*" AND OBESITY |
| "social venture* AND "indoor farm*" |
| "social venture* AND "urban farm*" |
| "social venture* AND farm* |
| "social venture*" AND "physical activity" |
| "social venture*" AND "weight loss" |
| "social venture*" AND diet |
| "social venture*" AND obesity |
| **Business Source Premier** |
| Social Entrepreneur* AND Physical Activity |
| Social Entrepreneur* AND "weight loss" |
| Social Entrepreneur* AND Diet |
| Social Entrepreneur* AND Obesity |
| Social Entrepreneur* AND Farm* |
| Social Entrepreneur* AND Indoor Farm* |
| Social Entrepreneur* AND Urban Farm* |
| Social Enterprise* AND Diet |
| Social Enterprise* AND Farm* |
| Social Enterprise* AND Indoor Farm* |
| Social Enterprise* AND Obesity |
| Social Enterprise* AND Physical Activity |
| Social Enterprise* AND Urban Farm* |
| Social Enterprise* AND Weight Loss |
| Social Innovat* AND Diet |
| Social Innovat* AND Farm* |
| Social Innovat* AND Indoor Farm* |
| Social Innovat* AND Obesity |
| Social Innovat* AND Physical Activity |
| Social Innovat* AND Urban Farm* |
| Social Innovat* AND Weight Loss |
| Social Venture* AND Diet |
| Social Venture* AND Farm* |
| Social Venture* AND Indoor Farm* |
| Social Venture* AND Obesity |
| Social Venture* AND Physical Activity |
| Social Venture* AND Urban Farm* |
| Social Venture* AND Weight Loss |
| **Factiva** |
| "Social Enterprise" AND "indoor farm" |
| "Social Enterprise" AND "physical activity" |
| "Social Enterprise" AND "urban farm" |
| "Social Enterprise" AND "weight loss" |
| "Social Enterprise" AND Diet |
| "Social Enterprise" AND farm |
| "Social Enterprise" AND Obesity |
| "Social Entrepreneur" AND "Farm*" |
| "Social Entrepreneur" AND "Indoor Farm*" |
| "Social Entrepreneur" AND "Physical Activity" |
| "Social Entrepreneur" AND "Urban Farm*" |
| "Social Entrepreneur" AND "Weight Loss" |
| "Social Entrepreneur" AND Diet |
| "Social Entrepreneur" AND Obesity |
| "Social innovation" AND "indoor farm" |
| "Social innovation" AND "physical activity" |
| "Social innovation" AND "urban farm" |
| "Social innovation" AND "weight loss" |
| "Social innovation" AND Diet |
| "Social innovation" AND farm |
| "social innovation" AND obesity |
| "social venture" AND "indoor farm*" |
| "social venture" AND "physical activity" |
| "social venture" AND "urban farm*" |
| "social venture" AND "weight loss" |
| "social venture" AND diet |
| "social venture" AND farm |
| "social venture" AND obesity |
| **PubMed** |
| (social enterprise*) AND (diet) |
| (social enterprise*) AND (farm*) |
| (social enterprise*) AND (indoor farm*) |
| (social enterprise*) AND (physical activity) |
| (social enterprise*) AND (urban farm*) |
| (social enterprise*) AND (weight loss) |
| (social enterprise*) AND Obesity |
| (social entrepreneur*) AND (diet) |
| (social entrepreneur*) AND (farm*) |
| (social entrepreneur*) AND (indoor farm*) |
| (social entrepreneur*) AND (physical activity) |
| (social entrepreneur*) AND (urban farm*) |
| (social entrepreneur*) AND (weight loss) |
| (social entrepreneur*) AND Obesity |
| (social innovat*) AND (diet) |
| (social innovat*) AND (farm*) |
| (social innovat*) AND (indoor farm*) |
| (social innovat*) AND (physical activity) |
| (social innovat*) AND (urban farm*) |
| (social innovat*) AND (weight loss) |
| (social innovat*) AND obesity |
| (social venture*) AND (diet) |
| (social venture*) AND (farm*) |
| (social venture*) AND (indoor farm*) |
| (social venture*) AND (physical activity) |
| (social venture*) AND (urban farm*) |
| (social venture*) AND (weight loss) |
| (social venture*) AND Obesity |

## Social Ventures Database (links last accessed Mar 2020)

| **No** | **Org name** | **Country of Origin** | **Diet/ nutrition** | **Physical activity** | **Healthy Food Sources Ex-Urban Farming** | **Urban Farming** | **Web Site** |
| --- | --- | --- | --- | --- | --- | --- | --- |
| 1 | The Community Grocer | Australia |  |  | * |  | https://www.thecommunitygrocer.com.au/impact |
| 2 | Capital Region Farmers Market | Australia |  |  | * |  | https://capitalregionfarmersmarket.com.au/ |
| 3 | Stephanie Alexander Kitchen Garden Foundation | Australia | * |  |  | * | https://www.kitchengardenfoundation.org.au/ |
| 4 | CERES Farm | Australia | * | * |  | * | https://ceres.org.au/ |
| 5 | Cultivating Community | Australia |  |  |  | * | http://www.cultivatingcommunity.org.au |
| 6 | Green Connect | Australia |  |  |  | * | https://green-connect.com.au/ |
| 7 | Playgroup Tasmania | Australia |  | * |  |  | https://playgrouptas.org.au/ |
| 8 | Cultivation Works | Australia |  |  |  | * | https://www.facebook.com/pg/cultivationworks/about/?ref=page_internal |
| 9 | Box Divvy | Australia |  |  | * |  | https://www.boxdivvy.com/ |
| 10 | Zambrero | Australia | * |  |  |  | <https://www.zambrero.com.au/> |
| 11 | Champion Life | Australia | * | * |  |  | <https://championlife.com.au/> |
| 12 | Food Connect Foundation | Australia |  |  | * |  | [http://foodconnectfoundation.org.au](http://foodconnectfoundation.org.au/) |
| 13 | Melbourne Food Hub | Australia |  |  |  | * | <https://melbournefoodhub.org.au/> |
| 14 | Kabuu | Australia |  |  |  | * | <http://www.kabuu.com.au/> |
| 15 | Perth Community Farm | Australia |  |  |  | * | <https://www.perthcommunityfarm.co.uk/the-farm-more> |
| 16 | Biofilta | Australia |  |  |  | * | [https://www.biofilta.com.au](https://www.biofilta.com.au/) |
| 17 | Peppergreen Farm | Australia |  |  | * |  | [https://peppergreenfarm.com.au](https://peppergreenfarm.com.au/) |
| 18 | The Paddock | Australia |  | * | * |  | [https://www.encompass-cs.org.au](https://www.encompass-cs.org.au/) |
| 19 | Yerrabingin | Australia |  |  |  | * | [https://www.yerrabingin.com.au](https://www.yerrabingin.com.au/) |
| 20 | Produce to the People | Australia | * |  |  | * | https://www.facebook.com/pg/producetothepeopletasmania/about/?ref=page_internal |
| 21 | Farmwall | Australia |  |  |  | * | <https://farmwall.com.au/> |
| 22 | The Farm Byron Bay | Australia | * |  | * |  | https://thefarm.com.au/contactus |
| 23 | Food Ladder | Australia |  |  | * |  | <https://foodladder.org/> |
| 24 | Ugrow Gardens Australia | Australia |  |  |  | * | <https://www.ugrowgardens.com/> |
| 25 | Irupana Andean Organic Food. S.R.L | Bolivia | * |  |  |  | http://www.irupanabio.com/ |
| 26 | Cidades Sem Fome Cities without Hunger | Brazil | * | * |  | * | https://cidadessemfome.org/ |
| 27 | Movere Institute | Brazil | * | * |  |  | http://institutomovere.org.br/ |
| 28 | ParaKids | Bulgaria |  | * |  |  | https://www.facebook.com/parakids.bg/ |
| 29 | Growcer | Canada |  |  |  | * | https://www.thegrowcer.ca/our-story |
| 30 | Lufa Farms Inc | Canada |  |  |  | * | https://montreal.lufa.com/en/ |
| 31 | Sole Food Street Farms | Canada |  |  |  | * | https://solefoodfarms.com/ |
| 32 | Capsana (previously known as ACTI-MENU) | Canada | * | * |  |  | https://www.capsana.ca/ |
| 33 | THE STEADWARD CENTRE for Personal & Physical Achievement | Canada |  | * |  |  | https://www.ualberta.ca/steadward-centre/about/ |
| 34 | The SEED | Canada | * |  |  | * | https://www.theseedguelph.ca |
| 35 | Seed by Seed | Canada | * | * |  | * | http://www.seedbyseed.ca |
| 36 | Feed it Forward | Canada | * |  | * |  | https://feeditforward.ca/ |
| 37 | FoodShare Toronto | Canada | * |  |  | * | https://foodshare.net/ |
| 38 | The Stop | Canada | * |  | * |  | https://www.thestop.org/ |
| 39 | Cowichan Green Community | Canada |  |  |  | * | [https://cowichangreencommunity.org](https://cowichangreencommunity.org/) |
| 40 | Farm Bound | Canada |  |  | * |  | https://www.farmbound.ca/our-story |
| 41 | Sercovie | Canada | * | * |  |  | https://translate.googleusercontent.com/translate_c?depth=1&rurl=translate.google.com&sl=auto&sp=nmt4&tl=en&u=http://sercovie.org/sercovie&xid=17259,15700021,15700186,15700191,15700256,15700259,15700262,15700265,15700271,15700283&usg=ALkJrhjADpRDvwYdDtxbDXOuXFPVu2gV-A |
| 42 | Urban Roots London | Canada |  |  |  | * | [https://urbanrootslondon.ca](https://urbanrootslondon.ca/) |
| 43 | Farafena | Canada |  |  | * |  | [https://farafena.com](https://farafena.com/) |
| 44 | Ripple Farms | Canada |  |  |  | * | <http://ripplefarms.ca/> |
| 45 | Hope Blooms | Canada | * |  |  | * | [https://hopeblooms.ca](https://hopeblooms.ca/) |
| 46 | Goodly Foods | Canada | * |  |  |  | [https://hellogoodly.ca](https://hellogoodly.ca/) |
| 47 | Building Roots | Canada |  |  |  | * | <http://buildingroots.ca/> |
| 48 | LifeCycles Project | Canada | * |  |  | * | <https://lifecyclesproject.ca/> |
| 49 | Fresh Roots | Canada | * |  |  | * | <https://freshroots.ca/> |
| 50 | Potluck Catering | Canada | * |  |  |  | <https://potluckcatering.org/> |
| 51 | Loutet Farm & The Edible Garden Project | Canada |  |  |  | * | http://ediblegardenproject.com/loutet-farm/ |
| 52 | Good Food Box | Canada |  |  | * |  | <https://thegoodfoodbox.ca/> |
| 53 | Graines de Chefs | Canada | * |  |  |  | <https://www.grainesdechefs.ca/> |
| 54 | PlayCity | Canada |  | * |  |  | <https://www.playcityapp.com/> |
| 55 | Sky Harvest | Canada |  |  |  | * | <http://skyharvest.ca/> |
| 56 | The Food Pedallers | Canada |  |  |  | * | <http://foodpedalers.ca/> |
| 57 | Living Produce Aisle | Canada |  |  |  | * | <http://livingproduceaisle.com/> |
| 58 | Evergreen | Canada |  | * |  | * | <https://www.evergreen.ca/> |
| 59 | FitSpirit /Fillactive | Canada | * | * |  |  | https://www.fitspirit.ca/ |
| 60 | Recess Project | Canada |  | * |  |  | http://www.recessprojectcanada.com/ |
| 61 | Green Monday Group | China | * | * | * |  | https://greenmonday.org/ |
| 62 | Food Heroes | China | * |  |  |  | https://www.foodheroes.org/ |
| 63 | Shared Harvest | China | * |  | * |  | https://sharedharvest.cn/aboutuszh/2017/10/01/%E5%86%9C%E5%9C%BA%E7%AE%80%E4%BB%8B/ |
| 64 | FitForKids | Denmark | * | * |  |  | http://www.fitforkids.dk/en |
| 65 | Empower | Egypt |  | * |  |  | https://www.facebook.com/pg/empower.fa/about/?ref=page_internal |
| 66 | Siel Bleu | France |  | * |  |  | https://www.sielbleu.org/ |
| 67 | La Tablée des Chefs (Band of Chefs) | France | * |  | * |  | https://www.tableedeschefs.org/en/media-documentation/ |
| 68 | Nomadisch Grün / Prinzessinnengärten | Germany | * | * |  | * | https://prinzessinnengarten.net |
| 69 | Green Common | Hongkong, China | * |  |  |  | https://www.greencommon.com |
| 70 | Rooftop Republic | Hongkong, China |  |  |  | * | https://www.rooftoprepublic.com/ |
| 71 | City Farm | Hongkong, China |  |  |  | * | <http://www.cityfarm.hk/> |
| 72 | Wildroots Organic | Hongkong, China | * |  |  | * | https://wildrootsorganic.com/ |
| 73 | GUNA Organics | India | * |  |  |  | <http://gunaorganics.org/> |
| 74 | UrbanMali | India |  | * |  | * | <https://www.urbanmali.com/> |
| 75 | The Sristi Farm Academy | India |  |  | * |  | http://www.sristivillage.org/impact-intro/ |
| 76 | Vilfresh by Laymen Agro Ventures | India |  |  | * |  | https://www.facebook.com/pg/VILFRESH/about/?ref=page_internal |
| 77 | Farmizen | India |  |  | * |  | <https://www.farmizen.com/> |
| 78 | Edible Routes | India |  |  |  | * | <http://edibleroutes.com/> |
| 79 | Indian Superheroes | India |  |  |  | * | http://indiansuperheroes.com/#aboutus |
| 80 | iKheti | India |  |  |  | * | https://www.facebook.com/pg/iKheti/about/?ref=page_internal |
| 81 | MonkeyBox Food Tech | India | * |  |  |  | https://monkeybox.in/ |
| 82 | Cure.fit | India | * | * |  |  | https://www.cure.fit/ |
| 83 | NutrifyMyDiet | India | * |  |  |  | http://nutrifymydiet.com/ |
| 84 | FunDay Sports | India |  | * |  |  | http://fundaysports.in/ |
| 85 | GIY (Grow It Yourself) | Ireland | * |  |  | * | [https://giy.ie](https://giy.ie/) |
| 86 | Sailing into Wellness | Ireland |  | * |  |  | https://www.sailingintowellness.ie/contact-us/ |
| 87 | Meals4Health | Ireland | * |  |  |  | https://www.meals4health.ie/meals4health/#/contact-us |
| 88 | Table for Two | Japan | * |  |  |  | http://www.tablefor2.org/home |
| 89 | Urban Hijau | Malaysia |  | * |  | * | [https://www.uhijau.org](https://www.uhijau.org/) |
| 90 | BTOP Sport Academy | Malaysia |  | * |  |  | https://www.b-top.com.my/ |
| 91 | Xilinat | Mexico |  |  |  |  | https://www.xilinat.com |
| 92 | Delinutrición de México SA de CV | Mexico | * |  |  |  | http://www.delinutricion.com.mx/ |
| 93 | Clinicas del Azucar | Mexico | * |  |  |  | https://www.clinicasdelazucar.com/ |
| 94 | Kokkoyao Organics | Myanmar |  |  |  | * | https://kokkoyaorganics.com/pages/contact-us |
| 95 | Doh Eain | Myanmar |  | * |  | * | [https://www.doheain.com](https://www.doheain.com/) |
| 96 | The Oopoeh Foundation | Netherlands |  | * |  |  | https://translate.google.com/translate?sl=auto&tl=en&u=https%3A%2F%2Fwww.oopoeh.nl |
| 97 | Eat My Lunch | New Zealand | * |  |  |  | https://www.eatmylunch.nz/ourstory/ |
| 98 | Cultivate Christchurch (previously Agropolis) | New Zealand |  |  |  | * | https://www.pledgeme.co.nz/loans/35-cultivate-christchurch |
| 99 | Kia Puāwai Charity | New Zealand | * | * |  |  | <https://kiapuawai.org.nz/> |
| 100 | PATU AOTEAROA | New Zealand |  | * |  |  | [https://www.patunz.com](https://www.patunz.com/) |
| 101 | Estacion Vital | Nicaragua | * | * |  |  | https://www.estacionvital.com/ |
| 102 | Fresh Direct Nigeria | Nigeria |  |  |  | * | https://www.facebook.com/FreshDirectNG/ https://www.f6s.com/freshdirect |
|  | AGREA | Philippines |  |  |  | * | [http://www.agreaph.com](http://www.agreaph.com/) |
| 104 | Uproot Urban Farms | Philippines |  |  |  | * | <https://uproot.ph/> |
| 105 | Vitamimos | Portugal | * | * |  |  | http://www.vitamimos.pt/ |
| 106 | Blooming Bs | Saudi Arabia | * |  |  |  | https://media-ashoka.oiengine.com/attachments/3c87cef4-2ad2-4a00-95ad-6830b03cec73.pdf |
| 107 | ProAge | Singapore | * | * |  |  | http://www.proage.sg/ |
| 108 | Filos Community Services | Singapore | * | * |  |  | https://www.filos.sg/contact |
| 109 | Sky Urban Solutions Holding Pte Ltd / Sky Greens | Singapore |  |  |  | * | https://www.skygreens.com/ |
| 110 | Edible Garden City | Singapore | * |  |  | * | https://www.ediblegardencity.com/ |
| 111 | Citizen Farm | Singapore |  |  |  | * | https://www.citizenfarm.com.sg/ |
| 112 | Comcrop | Singapore |  |  |  | * | https://comcrop.com/ |
| 113 | Bollywood Veggies | Singapore | * | * | * |  | http://bollywoodveggies.com/contacts |
| 114 | Samsui Supplies | Singapore | * |  |  |  | <https://samsui.com.sg/> |
| 115 | UCook | South Africa | * |  | * |  | https://ucook.co.za/ |
| 116 | Abalimi Bezekhaya | South Africa |  |  |  | * | http://abalimibezekhaya.org.za |
| 117 | Sleekgeek | South Africa |  | * |  |  | [https://www.sleekgeek.co.za](https://www.sleekgeek.co.za/) |
| 118 | L’Ortiga (Nettles) Ecological Products, Limited (SL) | Spain | * |  |  | * | https://content.sciendo.com/view/journals/euco/10/3/article-p398.xml?lang=en |
| 119 | Carmen Pardo-Valcarce Foundation | Spain |  |  |  | * | <https://www.sindromedownvidaadulta.org/no15-octubre-2013/buenas-practicas-experiencias-proyectos-n15/la-alegria-de-la-huerta-rus-in-urbe-la-huerta-de-montecarmelo/> |
| 120 | Freja Inc. / Barnmorska Åsa Österberg | Sweden |  | * |  |  | http://www.asa-osterberg.se/ |
| 121 | odla ihop öråker | Sweden |  |  |  | * | http://www.odlaihop.se/ |
| 122 | Theptarin Lifestyle Intervention Center | Thailand |  |  |  |  | https://www.theptarin.com/en/about-us/LifestyleInterventionCenter/ |
| 123 | b-fit center Turkey Sirnak | Turkey |  | * |  |  | https://www.kiva.org/lend/1235909?minimal=false |
| 124 | YACOB | UAE | * |  |  |  | https://www.linkedin.com/company/yacob |
| 125 | GrowUp Urban Farms | UK | * |  |  | * | https://www.growup.org.uk |
| 126 | GrowUp Community Farms | UK | * | * |  | * | https://www.growup.community/what-we-do |
| 127 | Nouveau Wellbeing | UK |  | * |  |  | https://www.nouveauwellbeing.com |
| 128 | Jamie's Ministry of Food | UK | * |  |  |  | https://www.jamiesministryoffood.com |
| 129 | Food Nation | UK | * |  | * |  | https://www.foodnation.org/ |
| 130 | North Glasgow Community Food Initiative | UK | * | * |  | * | https://www.ngcfi.org.uk/ |
| 131 | Vitality Corporate Services Limited | UK |  | * |  |  | https://www.vitality.co.uk/ |
| 132 | Social Farms & Gardens | UK | * | * |  | * | https://www.farmgarden.org.uk |
| 133 | Growing Communities | UK | * |  | * |  | https://www.growingcommunities.org/ |
| 134 | Good Food in Greenwich | UK | * |  |  | * | http://www.goodfoodingreenwich.org |
| 135 | My Time Active (MEND) | UK | * |  |  |  | https://www.mytimeactive.co.uk/ |
| 136 | HENRY | UK | * | * |  |  | https://henry.org.uk/ |
| 137 | Ella's Kitchen | UK | * |  |  |  | https://www.ellaskitchen.co.uk |
| 138 | Ecocenter | UK | * | * | * |  | http://www.eco-centre.org.uk/ |
| 139 | Evolve | UK | * | * |  |  | [https://www.evolvesi.com](https://www.evolvesi.com/) |
| 140 | Can Cook | UK | * |  |  |  | http://www.cancook.co.uk/cancook-cic/ |
| 141 | Community Food Enterprise (CFE) Limited | UK |  |  | * |  | <https://www.c-f-e.org.uk/> |
| 142 | fruit to suit | UK | * |  |  |  | <https://www.fruittosuit.co.uk/> |
| 143 | FARM:London | UK |  |  |  | * | <http://farmlondon.weebly.com/our-farms.html> |
| 144 | Providence Row | UK |  |  |  | * | <https://www.providencerow.org.uk/> |
| 145 | May Project Gardens | UK | * | * |  | * | <https://www.mayproject.org/> |
| 146 | Sporting Age | UK |  | * |  |  | [https://www.sportingage.co.uk](https://www.sportingage.co.uk/) |
| 147 | Transition Extreme | UK |  | * |  |  | https://www.transition-extreme.com/about-us |
| 148 | Cracking Good Food | UK | * |  |  |  | <https://www.crackinggoodfood.org/> |
| 149 | Health Champions | UK | * |  |  |  | <http://www.health-champions.co.uk/18/About-Us> |
| 150 | North East Dance CIC | UK |  | * |  |  | <https://northeastdance.com/> |
| 151 | You can cook | UK | * |  |  | * | [www.youcancook.org.uk/](http://www.youcancook.org.uk/) |
| 152 | Get Out More | UK |  | * |  |  | <https://www.getoutmorecic.co.uk/> |
| 153 | GLL CIC | UK |  | * |  |  | https://www.gll.org/b2b/contact |
| 154 | The Wiggly Worm | UK | * |  |  |  | https://www.thewigglyworm.org.uk/contact |
| 155 | Cycle Training Wales | UK |  | * |  |  | https://www.cycletrainingwales.org.uk/ |
| 156 | Little Bites Cookery Schol | UK | * |  |  |  | https://www.littlebitescookery.com/contact |
| 157 | Rural Regeneration Unit | UK | * |  | * |  | [http://www.ruralregeneration.org.uk](http://www.ruralregeneration.org.uk/) |
| 158 | Dietwise | UK | * |  |  |  | <https://www.dietwise.co.uk/> |
| 159 | Rabble | UK |  | * |  |  | <https://joinrabble.com/> |
| 160 | Yorkshire Yoga | UK |  | * |  |  | <https://yorkshireyoga.co.uk/> |
| 161 | Buzz Lockleaze | UK | * |  |  | * | [http://www.buzzlockleaze.co.uk](http://www.buzzlockleaze.co.uk/) |
| 162 | My Little Picolo | UK | * |  |  |  | <https://www.mylittlepiccolo.com/> |
| 163 | BH Live | UK |  | * |  |  | <https://www.bhlive.org.uk/> |
| 164 | Places for People Leisure | UK |  | * |  |  | <https://www.placesleisure.org/> |
| 165 | Active Communities | UK |  | * |  |  | <https://www.activecommunities.co.uk/> |
| 166 | Let’s Get Sporty | UK |  | * |  |  | <https://letsgetsporty.com/> |
| 167 | Pro-Life Fitness Centre | UK |  | * |  |  | <https://prolifefitnesscentre.com/> |
| 168 | McLaren Leisure | UK |  | * |  |  | <http://www.mclarenleisure.co.uk/> |
| 169 | Legacy School Sport CIC | UK | * | * |  |  | <https://www.legacysport.co.uk/> |
| 170 | Ayecan Change | UK | * | * |  |  | [https://www.ayecanchange.info](https://www.ayecanchange.info/) |
| 171 | Sport For Confidence | UK |  | * |  |  | [https://www.sportforconfidence.com](https://www.sportforconfidence.com/) |
| 172 | SMILE Through Sport | UK |  | * |  |  | [http://www.smilethroughsport.com](http://www.smilethroughsport.com/) |
| 173 | Tees Active | UK |  | * |  |  | [https://www.teesactive.co.uk](https://www.teesactive.co.uk/) |
| 174 | Mytimeactive | UK | * | * |  |  | <https://www.mytimeactive.co.uk/> |
| 175 | FIT4LIFEOVER50 | UK |  | * |  |  | <http://www.fit4lifeover50.co.uk/> |
| 176 | Edinburgh Leisure | UK |  | * |  |  | [https://www.edinburghleisure.co.uk](https://www.edinburghleisure.co.uk/) |
| 177 | Blackhill's Growing | UK |  | * |  | * | https://stpaulsyouthforum.co.uk/blackhills-growing/ |
| 178 | Wiltshire Farm Foods | UK | * |  |  |  | [https://www.wiltshirefarmfoods.com](https://www.wiltshirefarmfoods.com/) |
| 179 | Oddbox | UK |  |  | * |  | <https://www.oddbox.co.uk/> |
| 180 | Boxxfresh | UK | * |  | * |  | <https://www.boxxfresh.com/> |
| 181 | Abel & Cole | UK |  |  | * |  | https://www.abelandcole.co.uk/about-us |
| 182 | Cyrenians | UK | * |  | * |  | <https://cyrenians.scot/> |
| 183 | Velocity Café and Bicycle Workshop | UK |  | * | * |  | [http://velocitylove.co.uk](http://velocitylove.co.uk/) |
| 184 | Rooted in Hull | UK |  |  |  | * | http://www.rootedinhull.org.uk/contact1 |
| 185 | Cardiff Salad Garden | UK | * |  |  | * | <http://cardiffsaladgarden.co.uk/> |
| 186 | Maymessy | UK | * |  |  |  | <https://www.maymessy.com/> |
| 187 | Local Greens | UK | * |  |  |  | <https://www.localgreens.org.uk/> |
| 188 | The Community Farm | UK | * |  | * |  | <https://www.thecommunityfarm.co.uk/> |
| 189 | West Lothian Bike Library | UK |  | * |  |  | <https://www.wlbikelibrary.co.uk/> |
| 190 | Tablehurst Farm | UK | * |  | * |  | http://tablehurst.farm/ |
| 191 | Eves Hill Veg Co | UK |  |  | * |  | [https://eveshillvegco.com](https://eveshillvegco.com/) |
| 192 | Bridge Wellness Gardens | UK |  |  | * |  | [https://bwgardens.co.uk](https://bwgardens.co.uk/) |
| 193 | Spitalfields City Farm | UK |  | * | * |  | <https://www.spitalfieldscityfarm.org/> |
| 194 | Local Food Links | UK | * |  |  |  | http://www.localfoodlinks.org.uk/contact-us/ |
| 195 | Pennine Lancashire Community Farm | UK | * |  | * |  | <https://www.penninelancashirecommunityfarm.org/> |
| 196 | Stevenage Leisure Limited | UK |  | * |  |  | [https://www.sll.co.uk](https://www.sll.co.uk/) |
| 197 | Urban Growth | UK |  | * |  | * | <https://urbangrowth.london/> |
| 198 | Little Kitchen Magicians | UK | * |  |  |  | https://www.littlekitchenmagicians.com/ |
| 199 | Swansea Community Farm | UK |  | * | * |  | <https://www.swanseacommunityfarm.org.uk/> |
| 200 | Bosavern Community Farm | UK |  |  | * |  | <http://www.bosaverncommunityfarm.org.uk/> |
| 201 | Cultivate Oxford | UK |  |  | * |  | https://cultivateoxford.org/contact |
| 202 | Horton Community Farm | UK |  | * | * |  | [http://hcf.org.uk](http://hcf.org.uk/) |
| 203 | Cultivate London | UK |  |  |  | * | <https://cultivatelondon.org/> |
| 204 | Sutton Community Farm | UK |  |  | * |  | <http://suttoncommunityfarm.org.uk/> |
| 205 | Ecocentre Community Care Farm | UK | * | * | * |  | [http://www.eco-centre.org.uk](http://www.eco-centre.org.uk/) |
| 206 | Squash | UK | * |  |  | * | http://squashliverpool.co.uk/home |
| 207 | Wilson Stuart Active Society CIC | UK |  | * |  |  | [http://www.wsactivesociety.co.uk](http://www.wsactivesociety.co.uk/) |
| 208 | Fordhall Organic Farm | UK |  | * | * |  | <https://www.fordhallfarm.com/> |
| 209 | Whole School Meals | UK | * |  |  |  | <http://wholeschoolmeals.co.uk/> |
| 210 | Growing Well | UK |  | * | * |  | <https://growingwell.co.uk/> |
| 211 | C3 Collaborating for Health (C3) | UK | * | * |  |  | https://www.c3health.org/ |
| 212 | HealthyKart Community Inc | UK | * |  |  |  | http://healthykart.net/ |
| 213 | Mogo Lifelong Fitness | UK |  | * |  |  | https://www.jbs.cam.ac.uk/faculty-research/centres/social-innovation/cambridge-social-ventures/our-ventures/mogo-lifelong-fitness/ |
| 214 | EduMove | UK |  | * |  |  | http://www.edumove.co.uk/ |
| 215 | Boing Kids Ltd | UK |  | * |  |  | http://www.boingkids.co.uk/ |
| 216 | Sports Key | UK |  | * |  |  | https://sportskey.co.uk/ |
| 217 | SPARC | UK |  | * |  |  | https://www.sparcsport.com/ |
| 218 | Happy Go Cooking | UK | * |  |  |  | http://www.happygocooking.org/ |
| 219 | Bespoke Biking | UK |  | * |  |  | https://bespokebiking.com/ |
| 220 | Run An Empire | UK |  | * |  |  | http://www.runanempire.com/ |
| 221 | OurPath | UK | * |  |  |  | https://www.ourpath.co.uk/ |
| 222 | Vi- Ability | UK |  | * |  |  | http://www.vi-ability.org/ |
| 223 | MoveMe Dance | UK |  | * |  |  | <http://www.movemedance.co.uk/> |
| 224 | Growing Better CIC | UK |  |  |  | * | <http://growingbetter.mystrikingly.com/> |
| 225 | SEEDS NC | USA | * | * |  | * | http://www.seedsnc.org |
| 226 | The Produce Box | USA | * |  | * |  | https://theproducebox.com/contact-us/ |
| 227 | The Farmery | USA |  |  |  | * | https://www.facebook.com/pg/thefarmery/about/?ref=page_internal |
| 228 | Fitbit | USA |  | * |  |  | https://www.fitbit.com/sg/about |
| 229 | Let Kids Play | USA |  | * |  |  | https://www.facebook.com/pg/Let-Kids-Play-56755461579/about/?ref=page_internal |
| 230 | KaBOOM! | USA |  | * |  |  | https://kaboom.org/about_kaboom |
| 231 | Lose it! | USA | * | * |  |  | https://www.loseit.com/about/ |
| 232 | Calorie King | USA | * | * |  |  | https://www.calorieking.com/interactive-tools/ |
| 233 | Nutrisystem Inc | USA | * | * |  |  | https://www.nutrisystem.com/jsps_hmr/about/index.jsp |
| 234 | Walking Spree | USA |  | * |  |  | https://www.walkingspree.com |
| 235 | WW International | USA | * | * | * |  | https://www.weightwatchers.com/us/impact-manifesto |
| 236 | Exergame Fitness | USA |  | * |  |  | https://www.exergamefitness.com/contact/ |
| 237 | Motion Fitness | USA |  | * |  |  | https://www.motionfitness.com/contact-us-a/131.htm |
| 238 | Runkeeper | USA |  | * |  |  | https://runkeeper.com/ |
| 239 | Growing Gardens | USA | * | * |  | * | http://www.growing-gardens.org/contact/ |
| 240 | Community Servings | USA | * |  | * |  | https://www.servings.org/about-us/ |
| 241 | Conway Locally Grown | USA | * |  | * |  | https://conway.locallygrown.net/welcome |
| 242 | Sambazon | USA | * |  | * |  | https://www.sambazon.com/ |
| 243 | Organic Valley Coop | USA | * |  | * |  | https://www.organicvalley.coop/about-us/our-humble-history/ |
| 244 | Stonyfield | USA | * |  | * |  | https://www.stonyfield.com |
| 245 | FNV | USA | * |  |  |  | https://fnv.com/about/ |
| 246 | BOKS | USA | * | * |  |  | https://www.bokskids.org/ |
| 247 | Omada Health | USA | * | * |  |  | https://www.omadahealth.com/ |
| 248 | PowerUp | USA | * | * |  |  | https://www.powerup4kids.org/Home |
| 249 | School Lunch Initiative | USA | * | * |  | * | http://www.schoollunchinitiative.org/index.html |
| 250 | The Edible Schoolyard Project | USA | * |  |  | * | https://edibleschoolyard.org |
| 251 | Salvation Farms | USA | * |  | * |  | https://www.salvationfarms.org/our-work/ |
| 252 | Bon Appétit Management Company | USA | * |  |  |  | http://www.bamco.com/ |
| 253 | play:groundNYC | USA |  | * |  |  | https://play-ground.nyc/ |
| 254 | Zenger Farm | USA | * | * | * |  | https://zengerfarm.org/ |
| 255 | Revolution Foods | USA | * |  | * |  | https://www.revolutionfoods.com/ |
| 256 | Mother Wit Maternity & Consulting Services | USA | * | * |  |  | https://www.motherwitmaternity.com/about |
| 257 | Stockbox | USA | * |  | * |  | http://stockboxgrocers.com/about/ |
| 258 | City Health Works | USA |  |  |  |  | https://cityhealthworks.com/ |
| 259 | R&G Family Grocers / Healthy Community Store initiative | USA | * |  | * |  | https://www.tulsarealgoodfood.org/ |
| 260 | Montana Roots | USA | * |  |  | * | http://www.montanaroots.org/ |
| 261 | Gateway Greening | USA | * | * |  | * | http://www.gatewaygreening.org/ |
| 262 | Gardeneers | USA | * |  |  | * | http://gardeneers.org/ |
| 263 | Curriculum of Cuisine | USA | * |  |  |  | https://www.facebook.com/pg/The-Curriculum-of-Cuisine-1375683872676017/about/?ref=page_internal |
| 264 | Green Village Initiative | USA | * | * |  | * | https://www.gogvi.org/ |
| 265 | Healthy Foods for Healthy Kids | USA | * |  |  | * | https://healthyfoodsforhealthykids.org/ |
| 266 | Chop Chop Kids | USA | * |  |  |  | https://www.chopchopfamily.org/press-room |
| 267 | Nourish Wellness | USA | * |  |  | * | http://www.nourish-wellness.org/ |
| 268 | Healthy Fare for Kids | USA | * |  | * |  | https://www.healthyfareforkids.com/ |
| 269 | Massachusetts Avenue Project | USA | * |  |  | * | https://www.mass-ave.org/ |
| 270 | TOCA - Tohono O'odham Community Action | USA | * |  | * |  | https://www.facebook.com/pg/TOCA-Tohono-Oodham-Community-Action-137202393001616/about/?ref=page_internal |
| 271 | Project GROWS | USA | * |  | * |  | https://www.projectgrows.org/ |
| 272 | Backyard Growers | USA | * | * |  | * | http://www.backyardgrowers.org/ |
| 273 | Grow Dat Youth Farm | USA | * |  | * |  | https://growdatyouthfarm.org/ |
| 274 | City Schoolyard Garden | USA | * | * |  | * | https://www.cityschoolyardgarden.org/ |
| 275 | Green Mountain Farm to School | USA | * |  |  | * | http://greenmountainfarmtoschool.org/programs/farm-to-school/ |
| 276 | Valley Food and Farm | USA | * |  | * |  | https://vitalcommunities.org/valleyfoodfarm/ |
| 277 | Food Connects | USA | * |  | * |  | https://www.foodconnects.org/ |
| 278 | Youth Farm | USA | * |  |  | * | http://youthfarmmn.org/ |
| 279 | Bright Spot Ventures | USA |  |  |  | * | https://www.brightspotfarms.org/press |
| 280 | Conscious Connections, Inc | USA | * |  |  | * | https://www.facebook.com/pg/consciousconnectinc/about/?ref=page_internal |
| 281 | Shelburne Farms | USA | * |  | * |  | https://shelburnefarms.org |
| 282 | Isles | USA | * | * |  | * | <https://isles.org/> |
| 283 | Common Threads | USA | * |  |  |  | http://www.commonthreads.org |
| 284 | Midwest Food Connection | USA | * |  |  |  | https://www.midwestfoodconnection.org/ |
| 285 | Green Bronx Machine | USA | * |  |  | * | https://greenbronxmachine.org/ |
| 286 | Cooking with Kids | USA | * |  |  |  | https://cookingwithkids.org/ |
| 287 | Wellness in the Schools | USA | * | * |  |  | http://www.wellnessintheschools.org |
| 288 | DC Greens | USA | * |  |  | * | https://www.dcgreens.org/about |
| 289 | Washington Youth Garden | USA | * | * |  | * | <http://cityblossoms.org/> |
| 290 | Recipe for Success | USA | * |  |  | * | <https://www.recipe4success.org/press/what-theyre-saying.html> |
| 291 | Chef Ann Foundation | USA | * |  |  |  | http://www.chefannfoundation.org/ |
| 292 | Reap Food Group | USA | * |  | * |  | http://reapfoodgroup.org/ |
| 293 | Community GroundWorks | USA | * | * |  | * | http://www.communitygroundworks.org |
| 294 | Tilth Alliance | USA | * | * |  | * | <http://www.tilthalliance.org/> |
| 295 | Garden-Raised Bounty (GRuB) | USA | * |  |  | * | https://www.goodgrub.org/ |
| 296 | Tricycle Urban Ag Culture | USA | * |  |  | * | https://tricycleurbanag.org/ |
| 297 | Real Food for Kids’ | USA | * |  |  | * | https://www.realfoodforkids.org/ |
| 298 | Local Food Hub | USA | * |  |  | * | https://www.localfoodhub.org/ |
| 299 | Arcadia Center for Sustainable Food and Agriculture | USA | * |  |  | * | http://www.arcadiafood.org |
| 300 | The Center for an Agricultural Economy | USA | * |  | * |  | https://hardwickagriculture.org/about/our-impact |
| 301 | Youth Garden Project | USA | * |  |  | * | https://www.youthgardenproject.org |
| 302 | The Green Urban Lunch Box | USA | * |  |  | * | https://www.thegreenurbanlunchbox.com/ |
| 303 | Purple Asparagus | USA | * |  |  |  | https://purpleasparagus.org/ |
| 304 | Seeds of Solidarity Farm | USA | * |  |  | * | https://seedsofsolidarity.org |
| 305 | Athens Land Trust | USA |  |  |  | * | http://athenslandtrust.org/about/ |
| 306 | Boise Urban Garden School | USA | * | * |  | * | https://www.boiseurbangardenschool.org/ |
| 307 | Florida Introduces Physical Activity and Nutrition to Youth (FLIPANY) | USA | * | * |  |  | https://flipany.org/about-flipany/ |
| 308 | Lifelab | USA | * |  |  | * | https://www.lifelab.org/ |
| 309 | Acta Non Verba: Youth Urban Farm Project (ANV) | USA | * | * |  | * | https://anvfarm.org/mission/ |
| 310 | Malama Kaua’i | USA | * |  |  | * | http://www.malamakauai.org/ |
| 311 | Kōkua Hawai‘i Foundation | USA | * | * |  | * | https://www.kokuahawaiifoundation.org/programs |
| 312 | Schoolyard Roots (formerly Druid City Garden Project) | USA | * |  |  | * | https://schoolyardroots.org/ |
| 313 | EAT South | USA | * |  |  | * | https://www.eatsouth.org/ |
| 314 | Community Foodworks | USA | * |  |  | * | https://www.community-foodworks.org |
| 315 | Oxbow Farm & Conservation Center | USA | * |  | * |  | http://www.oxbow.org/about/ |
| 316 | Harlem Grown | USA | * | * |  | * | http://www.harlemgrown.org/sponsors |
| 317 | Wellspring Inc | USA | * |  | * |  | http://www.wellspringinc.org/ |
| 318 | DC Central Kitchen | USA | * |  | * |  | https://dccentralkitchen.org/ |
| 319 | The Nashville Food Project | USA | * |  |  | * | https://www.thenashvillefoodproject.org/ |
| 320 | Oldways | USA | * |  |  |  | https://oldwayspt.org |
| 321 | The Cooking Project | USA | * |  |  |  | http://thecookingproject.org/ |
| 322 | E.A.T. Foundation | USA | * |  | * |  | https://www.eatogether.org/ |
| 323 | Feed Communities | USA | * | * |  | * | https://www.feedcommunities.org/ |
| 324 | FRESH New London | USA | * |  |  | * | https://www.freshnewlondon.org/about-us |
| 325 | Fresh Future Farm | USA | * |  |  | * | https://www.freshfuturefarm.org/about-us |
| 326 | Springfield Community Gardens | USA | * |  |  | * | https://www.facebook.com/pg/SpringfieldCommunityGardens/about/?ref=page_internal |
| 327 | The Urban Farming Institute of Boston | USA | * |  |  | * | http://www.urbanfarminginstitute.org |
| 328 | Vertical Harvest of Jackson Hole | USA | * |  |  | * | https://www.verticalharvestjackson.com/ |
| 329 | Mid-Ohio Foodbank / URBAN FARMS OF CENTRAL OHIO | USA | * |  |  | * | https://www.midohiofoodbank.org/ |
| 330 | Vegas Roots Community Garden | USA |  |  |  | * | http://vegasroots.org/ |
| 331 | Urban Roots | USA | * |  |  | * | <https://www.urgc.org/> |
| 332 | The Big Garden | USA | * |  |  | * | https://biggarden.org/leadership |
| 333 | Community Crops | USA | * |  |  | * | http://communitycrops.org/ |
| 334 | City Sprouts / Omaha Sprouts | USA | * |  |  | * | https://www.omahasprouts.org/annual-report |
| 335 | Urban Harvest STL | USA | * |  |  | * | https://www.urbanharveststl.org/ |
| 336 | Helena Community Gardens | USA | * |  |  | * | http://helenagardens.org/about/ |
| 337 | Atlanta Harvest | USA |  |  |  | * | https://atlantaharvest.com/about-us/ |
| 338 | Willamette Farm and Food Coalition | USA | * |  | * |  | http://willamettefarmandfood.org/our-work/ |
| 339 | Southside Community Land Trust (SCLT | USA | * |  |  | * | https://www.southsideclt.org/about-us/ |
| 340 | Project Feed The Hood | USA | * |  |  | * | http://www.projectfeedthehood.org/ |
| 341 | Urban Adamah | USA | * |  |  | * | https://www.urbanadamah.org/about/ |
| 342 | Philadelphia Orchard Project | USA | * |  |  | * | https://www.phillyorchards.org/ |
| 343 | Eco City Farms | USA | * |  |  | * | http://www.ecoffshoots.org |
| 344 | Sprout NOLA | USA | * |  |  | * | https://www.sproutnolafarm.org/about-us |
| 345 | Common Good City Farm | USA | * |  |  | * | https://www.commongoodcityfarm.org/ |
| 346 | Neighborhood Farm Initiative | USA | * |  |  | * | http://neighborhoodfarminitiative.org/our-mission/ |
| 347 | Rooftop Roots | USA |  |  |  | * | http://rooftoproots.org/ |
| 348 | Nuestras Raices | USA | * |  |  | * | https://nuestras-raices.org/ |
| 349 | Changelab Solutions | USA |  |  |  |  | https://www.changelabsolutions.org/ |
| 350 | Sweet Water Foundation | USA |  |  |  | * | https://www.sweetwaterfoundation.com |
| 351 | City Fruit | USA |  |  |  | * | https://www.cityfruit.org/ |
| 352 | Wasatch Community Gardens | USA | * |  |  | * | https://wasatchgardens.org/ |
| 353 | Plant Chicago | USA |  |  |  | * | https://plantchicago.org |
| 354 | Grow Pittsburgh | USA |  |  |  | * | https://www.growpittsburgh.org |
| 355 | Greensgrow | USA |  |  |  | * | https://www.greensgrow.org/ |
| 356 | Detroit Black Community Food Security Network (DBCFSN) | USA | * |  |  | * | https://www.dbcfsn.org/mission-vision-values |
| 357 | Green City Growers | USA |  |  |  | * | https://greencitygrowers.com/projects/ |
| 358 | CommonWealth Urban Farms | USA |  |  |  | * | http://commonwealthurbanfarms.com/ |
| 359 | City Roots | USA | * |  |  | * | https://cityroots.org/ |
| 360 | Urban Oasis Project | USA | * |  |  | * | http://www.urbanoasisproject.org/ |
| 361 | Common Ground | USA | * | * |  | * | http://www.commongroundct.org/ |
| 362 | Denver Urban Gardens DUG | USA | * | * |  | * | https://dug.org |
| 363 | City Slicker Farms | USA | * |  |  | * | http://www.cityslickerfarms.org/about.php |
| 364 | Friendship Gardens | USA | * |  |  | * | http://friendship-gardens.org/ |
| 365 | Ho`oulu Pacific | USA | * |  |  | * | http://www.hooulupacific.org |
| 366 | Community Foods Market (previously People's Grocery) | USA | * |  | * |  | https://www.communityfoodsmarket.com/ |
| 367 | Local Matters | USA | * |  | * |  | https://www.local-matters.org/our-work |
| 368 | Playworks | USA |  | * |  |  | https://www.playworks.org/ |
| 369 | Livongo | USA | * | * |  |  | https://www.livongo.com/ |
| 370 | Brooklyn Grange | USA | * |  |  | * | https://www.brooklyngrangefarm.com/ |
| 371 | Born Juice | USA | * |  |  |  | https://www.instagram.com/bornjuice/ |
| 372 | Planting Justice | USA | * |  |  | * | http://plantingjustice.org/ |
| 373 | Phat Beets Produce | USA | * |  |  | * | https://www.phatbeetsproduce.org/ |
| 374 | Windy City Harvest Youth Farm | USA | * |  |  | * | https://www.chicagobotanic.org/urbanagriculture/youthfarm |
| 375 | 412 Food Rescue | USA | * |  | * |  | https://412foodrescue.org/programs/food-rescue/about-food-rescue/ |
| 376 | Black Urban Gardeners and Farmers of Pittsburgh Co-Op (BUGs) | USA | * |  |  | * | https://bugfpc.com/about-us/ |
| 377 | Gardener's Supply Company | USA | * | * |  | * | https://www.gardeners.com/ |
| 378 | Square Roots | USA |  |  |  | * | https://squarerootsgrow.com/program/ |
| 379 | So All May Eat Inc | USA | * |  |  | * | https://www.soallmayeat.org/ |
| 380 | Mala ʻAi ʻOpio Community Food Systems Initiative | USA |  |  |  | * | https://www.maoorganicfarms.org |
| 381 | America’s Grow-a-Row’s | USA | * | * | * |  | https://www.americasgrowarow.org/ |
| 382 | 18 Reasons | USA | * |  |  |  | https://18reasons.org/ |
| 383 | Ceres Community Project | USA | * |  |  | * | https://www.ceresproject.org/# |
| 384 | Farmer Foodshare | USA | * |  | * |  | http://www.farmerfoodshare.org |
| 385 | Working Landscapes | USA | * |  | * |  | http://workinglandscapesnc.org |
| 386 | Back to the Roots | USA | * |  |  | * | https://backtotheroots.com/ |
| 387 | Garden Fresh Farms | USA |  |  |  | * | http://www.gardenfreshfarms.org/ |
| 388 | Florida Organic Growers | USA | * |  | * |  | https://foginfo.org/ |
| 389 | Community Farmers Markets | USA | * |  | * |  | https://cfmatl.org/ |
| 390 | Farmer D Consulting | USA |  |  |  | * | https://farmerdconsulting.com/ |
| 391 | Hungry Harvest | USA |  |  | * |  | https://www.hungryharvest.net/ |
| 392 | Fresh Food Generation | USA | * |  | * |  | https://www.freshfoodgeneration.com/ |
| 393 | The Oasis Institute | USA | * | * |  |  | https://www.oasisnet.org/ |
| 394 | PHRQL | USA | * |  |  |  | https://phrql.com/ |
| 395 | Naturebox | USA | * |  |  |  | https://naturebox.com |
| 396 | Veterans to Farmers | USA |  |  |  | * | https://www.veteranstofarmers.org/ |
| 397 | Sicangu Food Sovereignty Initiative | USA | * | * | * |  | https://www.sicangucdc.org/keya-wakpala-gardens |
| 398 | GrowNYC | USA |  | * |  | * | https://www.grownyc.org/ |
| 399 | Farm to City | USA |  |  | * |  | https://farmtocity.org/ |
| 400 | Urban Tree Connection | USA | * |  |  | * | http://www.urbantreeconnection.org |
| 401 | St. Christopher’s Foundation for Children (SCFC) | USA | * |  |  |  | https://scfchildren.org |
| 402 | The Food Trust | USA | * |  | * |  | http://thefoodtrust.org/what-we-do/research |
| 403 | ReVision Urban Farm | USA |  |  |  | * | https://www.vpi.org/revision/ |
| 404 | Recirculating Farms Coalition | USA |  |  |  | * | http://www.recirculatingfarms.org/ |
| 405 | Urban Growers Collective | USA | * |  |  | * | https://urbangrowerscollective.org |
| 406 | Martha's Table | USA | * |  | * |  | https://marthastable.org |
| 407 | The Michigan Urban Farming Initiative | USA |  |  |  | * | https://www.miufi.org/ |
| 408 | Red Tomato | USA |  |  | * |  | https://redtomato.org/ |
| 409 | Farmraiser | USA | * |  | * |  | https://www.farmraiser.com/ |
| 410 | The Cookbook Project | USA | * |  |  |  | https://thecookbookproject.org/ |
| 411 | New Orleans Food & Farm Network | USA | * |  |  | * | https://www.noffn.org/ |
| 412 | Sustainable Food Center | USA | * |  |  | * | https://sustainablefoodcenter.org/ |
| 413 | Ridge to Reef Farm | USA |  |  | * |  | http://www.ridge2reef.org/# |
| 414 | Bedford Stuyvesant Restoration Corporation Farm to Early Care Program | USA | * | * | * |  | https://restorationplaza.org |
| 415 | Ardmore Institute of Health (AIH) | USA | * | * |  |  | https://www.ardmoreinstituteofhealth.org |
| 416 | About Ian's Elevation Brands, LLC. | USA | * |  |  |  | http://iansnaturalfoods.com/ |
| 417 | Kitchens For Good | USA | * |  | * |  | https://kitchensforgood.org/ |
| 418 | Village Gardens | USA |  |  |  | * | http://www.villagegardens.org |
| 419 | Appetite for Change | USA | * |  | * |  | https://appetiteforchangemn.org/ |
| 420 | Earthworks Urban Farm of Capuchin Soup Kitchen | USA | * |  | * |  | https://www.cskdetroit.org/earthworks/ |
| 421 | The Food Project | USA | * |  |  | * | https://thefoodproject.org/ |
| 422 | DC Urban Greens | USA | * |  |  | * | https://www.dcurbangreens.org/ |
| 423 | Rebel Ventures | USA | * |  |  |  | https://www.rvcrew.com/ |
| 424 | Grow Where You Are LLC | USA |  |  |  | * | https://www.growwhereyouare.farm |
| 425 | Maitufoods | USA | * |  |  |  | https://www.maitufoods.com/ |
| 426 | SweetRoots Kitchen | USA | * |  |  |  | https://www.sweetrootskitchen.org/contact |
| 427 | Self-Management Resource Center, | USA |  |  |  |  | https://www.selfmanagementresource.com/ |
| 428 | This Saves Lives | USA | * |  |  |  | https://thissaveslives.com/pages/faq |
| 429 | Loco'l / Locol | USA | * |  |  |  | https://www.welocol.com/ https://www.indiegogo.com/projects/locol-revolutionary-fast-food?mod=article_inline#/ |
| 430 | California FreshWorks | USA |  |  | * |  | http://www.cafreshworks.com/ |
|  | Active Science | USA |  | * |  |  | https://activescienceforkids.org/research/ |
| 432 | CommunityGrows | USA | * | * |  | * | https://www.communitygrows.org/ |
| 433 | Garden School Foundation | USA | * | * |  | * | https://gardenschoolfoundation.org/ |
| 434 | Groundwork Denver | USA | * | * |  | * | https://groundworkcolorado.org/ |
| 435 | Forge City Works | USA |  |  | * |  | https://www.forgecityworks.org/ |
| 436 | Sankofa Community Development Corporation | USA | * |  |  | * | http://sankofanola.org/ |
| 437 | Eden Place Nature Center / Fuller Park Community Development Corporation (FPCD) | USA |  | * |  | * | http://www.edenplacenaturecenter.org/ |
| 438 | Fan4Kids | USA | * | * |  |  | https://www.fan4kids.org |
| 439 | Figure Skating in Harlem | USA |  | * |  |  | https://figureskatinginharlem.org |
| 440 | Mill Creek Urban Farm | USA |  |  |  | * | https://www.millcreekurbanfarm.org/ |
| 441 | Greater Richmond Fit4Kids | USA |  | * |  |  | https://grfit4kids.org/ |
| 442 | Green Plate Special | USA | * |  |  | * | http://greenplatespecial.org |
| 443 | FortWhyte Farms | USA | * |  | * |  | https://www.fortwhytefarms.com/ |
| 444 | Roots to Harvest | USA | * |  |  | * | http://www.rootstoharvest.org/ |
| 445 | Seed and Roe (formerly Eden Works) | USA |  |  |  | * | https://seedandroe.com |
| 446 | Coastal Roots Farm | USA |  |  | * |  | https://coastalrootsfarm.org/ |
| 447 | Micro Farms LLC | USA |  |  |  | * | http://microfarmscolorado.com/contact-micro-farms/ |
| 448 | Fresh International Gardens | USA |  |  | * |  | https://www.cssalaska.org/blog/grow-north-farm-raiser/ |
| 449 | Urban Roots GR | USA | * |  |  | * | https://www.urbanrootsgr.org/ |
| 450 | Urban Roots ATX | USA |  |  |  | * | https://urbanrootsatx.org/youth-in-the-community/ |
| 451 | Growing Home Inc (formerly Wood Street Urban Farm) | USA |  |  |  | * | http://growinghomeinc.org/ |
| 452 | Lawrence Community Gardens | USA | * |  |  | * | http://lawrencecommunitygardens.org/ |
| 453 | Huntington's Kitchen | USA | * |  |  |  | http://huntingtons-kitchen.org/contact-us/ |
| 454 | GreenWheel Food Hub | USA |  |  | * |  | http://www.greenwheelfoodhub.org/ |
| 455 | Focus Foods Inc | USA |  |  |  | * | http://www.focusfoodsinc.com/ |
| 456 | Alaska Seeds of Change | USA |  |  |  | * | https://acmhs.com/what-we-do/vocational-services/alaska-seeds-of-change/ |
| 457 | Ripe Revival | USA | * |  |  |  | https://www.riperevival.com/ |
| 458 | Freight Farms | USA |  |  |  | * | https://www.freightfarms.com |
| 459 | VH Hydroponics (VHH) | USA |  |  |  | * | https://vhhydroponics.com/ |
| 460 | M2M Community Foundation Nile Valley Aquaponics 100,000 Pound Food Project | USA | * |  |  | * | <http://nilevalleyaquaponics.com/> |
| 461 | Zeponic Farms | USA |  |  |  | * | http://www.zeponicfarms.com/mission-index-impact |
| 462 | Seeds of Change | USA | * |  |  | * | <https://www.seedsofchange.com/> |
| 463 | Hana Health | USA | * |  |  | * | http://hanahealth.org/about-us/contact-us/ |
| 464 | Green Leaf Learning Farm | USA | * |  |  | * | <https://www.knowledgequest.org/> |
| 465 | Bike Works | USA |  | * |  |  | <https://bikeworks.org/> |
| 466 | Asiya Sport | USA |  | * |  |  | <https://www.asiyasport.com/> |
| 467 | Chrysalis Center Inc | USA |  |  |  | * | <https://chrysaliscenterct.org/> |
| 468 | Iona Senior Services | USA | * | * |  |  | [https://www.iona.org](https://www.iona.org/) |
| 469 | American Council on Exercise (ACE) | USA | * | * |  |  | <https://www.acefitness.org/> |
| 470 | RecoveryPark | USA |  |  |  | * | <https://www.recoverypark.org/> |
| 471 | Bon Secours Community Works’ Urban Farm and Community Garden | USA |  |  |  | * | https://bonsecours.com/baltimore/about-us/newsroom/news/future-baltimore-launches-initiative-to-ease-food-deserts-in-west-baltimore |
| 472 | Poughkeepsie Farm Project | USA | * |  | * |  | <https://www.farmproject.org/> |
| 473 | East New York Farms! | USA | * |  |  | * | https://ucceny.org/enyf/ |
| 474 | New City Farm | USA | * |  | * |  | https://newcityneighbors.org/farm/ |
| 475 | Victory Garden Initiative | USA | * |  |  | * | [https://victorygardeninitiative.org](https://victorygardeninitiative.org/) |
| 476 | Raleigh City Farm | USA |  |  |  | * | [https://raleighcityfarm.org](https://raleighcityfarm.org/) |
| 477 | Farm School NYC | USA |  |  |  | * | [https://www.farmschoolnyc.org](https://www.farmschoolnyc.org/) |
| 478 | Evo Farm | USA |  |  |  | * | <http://www.evofarm.com/> |
| 479 | Cooking Matters | USA | * |  |  |  | <https://cookingmatters.org/> |
| 480 | SuperBetter | USA |  | * |  |  | <https://www.superbetter.com/> |
| 481 | Chartwells Schools | USA | * |  |  |  | <http://www.chartwellsschools.com/> |
| 482 | Karma.Farm (Farm to Clinic Project with Johns Hopkins Social Innovation Lab) | USA |  |  | * |  | <http://karma.farm/> |
| 483 | Urban Pastoral Co | USA |  |  |  | * | <http://www.urbanpastoral.co/> |
| 484 | Eat the Yard | USA |  |  |  | * | [http://www.eattheyard.net](http://www.eattheyard.net/) |
| 485 | Stone Barns Center for Food and Agriculture | USA | * |  | * |  | [https://www.stonebarnscenter.org](https://www.stonebarnscenter.org/) |
| 486 | Trailnet | USA |  | * |  |  | http://www.trailnet.org/ |
| 487 | h.u.m.a.n. (“Helping Unite Mankind And Nutrition”) | USA | * |  | * |  | http://www.healthyvending.com/ |
| 488 | Good Bowls | USA | * |  |  |  | https://eatgoodbowls.com/ |
| 489 | Healthy School Food Collaborative (HSFC) | USA | * |  |  |  | https://www.thehealthyschoolfoodcollaborative.com/ |
| 490 | YFDS(Youth & Families Determined to Succeed) | USA | * | * |  | * | https://www.yfds.org/ |
| 491 | Cubii | USA |  | * |  |  | http://www.mycubii.com/ |
| 492 | Everytable | USA | * |  | * |  | https://www.everytable.com/ |
| 493 | Perk Health | USA | * | * |  |  | http://www.perkhealth.me/ |
| 494 | Yoga Foster | USA |  | * |  |  | https://yogafoster.org/ |
| 495 | The Common Market | USA |  |  | * |  | https://www.thecommonmarket.org/ |
| 496 | Kinosol | USA | * |  | * |  | http://www.getkinosol.com/#savethethird |
| 497 | Scrumptfresh | USA | * |  | * |  | http://www.scrumptfresh.com/ |
| 498 | Virgin Pulse (formerly Blue Mesa Health) | USA |  | * |  |  | http://www.bluemesahealth.com/ |
| 499 | MEVO Mahwah Environmental Volunteers Organization, Inc. | USA | * |  | * |  | http://mevoearth.org/the-farm/ |
| 500 | Hartford Food Systems (The Grow Hartford Urban Farm) | USA | * |  |  | * | http://www.hartfordfood.org/programs/hartford-mobile-market/ |
| 501 | BK Farmyards | USA |  |  |  | * | http://bkfarmyards.com/about/ |
| 502 | Rimidi | USA |  |  |  |  | http://www.rimidi.com/ |
| 503 | Wellnesss Corporate Solutions | USA |  |  |  |  | https://www.wellnesscorporatesolutions.com/ |
| 504 | FoodCorps | USA | * |  | * |  | https://foodcorps.org/ |
| 505 | Footprints to Fitness | USA |  | * |  |  | https://www.footprintstofitness.com/ |
| 506 | Fitlot | USA |  | * |  |  | http://fitlot.org/ |
| 507 | The Healthy School Food Collaborative | USA | * |  | * |  | http://www.thsfc.com/ |
| 508 | Top Box Foods | USA |  |  | * |  | http://www.topboxfoods.com/ |
| 509 | Kurbo (now part of WW Weight Watchers) | USA | * | * |  |  | https://kurbo.com/about/ |
| 510 | Boundless Playgrounds® | USA |  | * |  |  | https://changingthepresent.org/collections/boundless-playgrounds |
| 511 | 4P FOODS | USA | * |  | * |  | http://4pfoods.com/how-it-works |
| 512 | Green Bridge Growers | USA | * |  |  | * | <https://www.greenbridgegrowers.org/> |
